# Centralized Hierarchical Coded Caching Scheme for Two-Layer Network

**DOI:** 10.3390/e27030316

**Published:** 2025-03-18

**Authors:** Kun Zhao, Jinyu Wang, Minquan Cheng

**Affiliations:** 1 Key Lab of Education Blockchain and Intelligent Technology, Ministry of Education, Guangxi Normal University, Guilin 541004, China; zk4079kfzs@163.com (K.Z.); chengqinshi@hotmail.com (M.C.); 2 School of Mathematics and Statistics, Guangxi Normal University, Guilin 541004, China

**Keywords:** coded caching, hierarchical networks, placement delivery array

## Abstract

This paper considers a two-layer hierarchical network, where a server containing *N* files is connected to $K_{1}$ mirrors and each mirror is connected to $K_{2}$ users. Each mirror and each user has a cache memory of size $M_{1}$ and $M_{2}$ files, respectively. The server can only broadcast to the mirrors, and each mirror can only broadcast to its connected users. For such a network, we propose a novel coded caching scheme based on two known placement delivery arrays (PDAs). To fully utilize the cache memory of both the mirrors and users, we first treat the mirrors and users as cache nodes of the same type; i.e., the cache memory of each mirror is regarded as an additional part of the connected users’ cache, then the server broadcasts messages to all mirrors according to a $K_{1}K_{2}$-user PDA in the first layer. In the second layer, each mirror first cancels useless file packets (if any) in the received useful messages and forwards them to the connected users, such that each user can decode the requested packets not cached by the mirror, then broadcasts coded subpackets to the connected users according to a $K_{2}$-user PDA, such that each user can decode the requested packets cached by the mirror. The proposed scheme is extended to a heterogeneous two-layer hierarchical network, where the number of users connected to different mirrors may be different. Numerical comparison showed that the proposed scheme achieved lower coding delays compared to existing hierarchical coded caching schemes at most memory ratio points.

## 1. Introduction

With the development of the Internet, video and social media traffic occupy the main part of the network, leading to serious congestion. Due to the unbalanced distribution of network traffic over time, caching technology has been proposed to alleviate network congestion. In [1], Maddah-Ali and Niesen (MN) proposed a coded caching scheme (referred to as the MN scheme) for the (K,M,N) caching system: a server containing *N* files is connected to *K* users through an error-free shared link, and each user has an isolated cache of size *M* files. A coded caching scheme operates in two phases. In the placement phase, each file is divided into *F* packets, some of which are stored in each user’s cache according to a certain strategy. The quantity *F* is referred to as the subpacketization. In the delivery phase, network coding techniques are utilized to combine the packets requested by multiple users into multicast messages for transmission, such that each user can rebuild their desired file. The worst-case normalized transmission amount in the delivery phase is referred to as the transmission load *R*. Coded caching realizes multicast transmission for multiple users with different demands, which can effectively reduce the transmission load compared to uncoded caching. The MN scheme is optimal under uncoded placement when K≤N [2,3,4], and is order optimal within a factor of 2 with respect to the information-theoretic lower bound [5]. However, the subpacketization of the MN scheme increases exponentially with the number of users. In order to reduce the subpacketization, Shanmugam et al. [6] proposed a grouping method, which divides the users into several groups and applies the MN scheme to each group. Yan et al. [7] proposed a combinatorial structure called a placement delivery array (PDA) to represent coded caching schemes. The MN scheme can be represented by a special class of PDA, which is referred to as the MN PDA. Based on the concept of PDA, several coded caching schemes were proposed in [7,8,9,10,11,12]. Other combinatorial structures, including linear block codes [13], special (6,3)-free hypergraphs [14], (r,t) Ruzsa-Szeméredi graphs [15], projective geometry [16], combinatorial designs [17], and rainbow structures [18], have also been utilized in the design of coded caching schemes.

### 1.1. Two-Layer Hierarchical Network Model and Related Works

**Figure 1:**
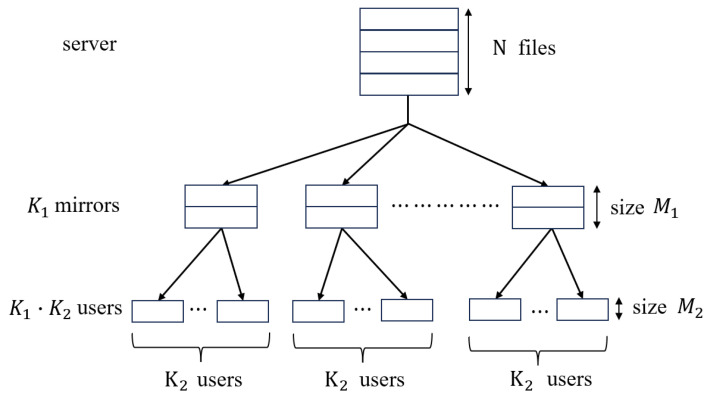
The two-layer hierarchical network from [19].

In practical applications, most caching systems consist of multiple layers. This paper considers the ($K_{1},K_{2},M_{1},M_{2},N$) two-layer hierarchical network proposed in [19], as shown in Figure 1. There is a single server connected to $K_{1}$ mirrors through an error-free broadcast link. Likewise, each mirror is connected to $K_{2}$ users, so there are $K=K_{1}K_{2}$ users in total. The server contains *N* equal-size files denoted by $W_{1},\cdots ,W_{N}$. Each mirror and each user have a cache memory of size $M_{1}$ and $M_{2}$ files, respectively. The *j*-th user attached to the *i*-th mirror is denoted by user (*i*, *j*), where $i\in \{1,2,\cdots ,K_{1}\}$, $j\in \{1,2,\cdots ,K_{2}\}$.

The network operates in two phases:

- **Placement Phase:** Each file $W_{n}$ is split into *F* packets with equal size, i.e., $W_{n}=\left\{W_{n,f}|f\in \left\{1,2,\cdots ,F\right\}\right\}$; then, the mirrors and users cache some packets of each file. Denote the content cached by the *i*-th mirror and user (i,j) by $M_{i}$ and $U_{\left(i,j\right)}$, respectively. In this phase, the server has no knowledge of the users’ future requests.
- **Delivery Phase:** Each user randomly requests a file from the server, assuming that user (i,j) requests the file $W_{d_{\left(i,j\right)}}$, the request is denoted by $d=(d_{\left(1,1\right)},\;d_{\left(1,2\right)},\cdots ,\;d_{\left(1,K_{2}\right)},\cdots ,\;$$d_{\left(K_{1},K_{2}\right)})$. The server broadcasts coded messages of total size $R_{1}$ files to all mirrors, and each mirror broadcasts coded messages of total size $R_{2}$ files to all its attached users, such that each user can rebuild their desired file. $R_{1}$ is referred to as the transmission load from the server to the mirrors, and $R_{2}$ is called the transmission load from each mirror to its attached users.

Notice that a parallel transmission may exist between the server and mirrors due to the orthogonal links between the two layers. Thus, in a two-layer hierarchical network, if the server and all mirrors concurrently send symbols through all transmission slots, then the corresponding coding delay is

$$T_{max}=\max\left\{R_{1},R_{2}\right\}.$$

If there exists a mirror which starts transmission after the server finishes its transmission, the coding delay is

$$T_{sum}=R_{1}+R_{2}.$$

The goal is to design coded caching schemes with the coding delay $T_{max}$ or $T_{sum}$ as small as possible.

For the two-layer hierarchical network, Nikhil Karamchandani et al., in [19], divided each file in the server into an α fraction and (1−α) fraction, and divided each user cache memory into a β fraction and (1−β) fraction. By applying the decentralized MN scheme to the two parts of the files separately, the achieved transmission loads are as follows:

$$R_{1}=\alpha \cdot K_{2}\cdot \;r_{d}\left(\frac{M_{1}}{\alpha },K_{1}\right)+\left(1-\alpha \right)\cdot r_{d}\left(\frac{\left(1-\beta \right)M_{2}}{\left(1-\alpha \right)},K_{1}K_{2}\right)$$

$$R_{2}=\alpha \cdot r_{d}\left(\frac{\beta M_{2}}{\alpha },K_{2}\right)+\left(1-\alpha \right)\cdot r_{d}\left(\frac{\left(1-\beta \right)M_{2}}{\left(1-\alpha \right)},K_{2}\right)$$

where

$$r_{d}\left(M,K\right)=\left\{\begin{array}{cc} \left(\frac{N}{M}-1\right)\left(1-{\left(1-\frac{M}{N}\right)}^{K}\right)\; & \mathrm{if}\;0<\frac{M}{N}<1,\\ K\; & \mathrm{if}\;\frac{M}{N}=0,\\ 0\; & \mathrm{otherwise}. \end{array}\right.$$

It was shown that $R_{1}$ and $R_{2}$ are simultaneously approximately minimized when $\alpha =\alpha^{*}$ and $\beta =\beta^{*}$, where

$$\left(\alpha^{*},\beta^{*}\right)=\left\{\begin{array}{cc} (\frac{M_{1}}{N},\frac{M_{1}}{N})\;\; & \mathrm{if}\;M_{1}+M_{2}K_{2}\geq N,\;0\leq M_{1}\leq \frac{N}{4},\\ (\frac{M_{1}}{M_{1}+M_{2}K_{2}},0)\;\; & \mathrm{if}\;M_{1}+M_{2}K_{2}<N,\\ (\frac{M_{1}}{N},\frac{1}{4})\;\; & \mathrm{if}\;M_{1}+M_{2}K_{2}\geq N,\;\frac{N}{4}<M_{1}\leq N. \end{array}\right.$$

When $\frac{M_{1}}{N}\in \left\{0,\frac{1}{K_{1}},\cdots ,\frac{K_{1}-1}{K_{1}},1\right\}$ and $\frac{M_{2}}{N}\in \left\{0,\frac{1}{K_{2}},\cdots ,\frac{K_{2}-1}{K_{2}},1\right\}$, Wang et al. [20] proposed a centralized coded caching scheme that leverages idle transmission time resources by constructing concurrent transmissions between the two layers, achieving the following maximum coding delay:

$$T_{max}=\alpha R_{s1}+\left(1-\alpha \right)R_{s2},$$

where

$$R_{s1}=r_{c}\left(\frac{M_{1}}{\alpha },K_{1}\right)r_{c}\left(\frac{\beta M_{2}}{\alpha },K_{2}\right)+\min\left(\frac{M_{1}}{\alpha N},1\right)r_{c}\left(\frac{\beta M_{2}}{\alpha },K_{2}\right),$$

$$R_{s2}=r_{c}\left(\frac{\left(1-\beta \right)M_{2}}{1-\alpha },K_{1}K_{2}\right),$$

and $r_{c}\left(M,K\right)=\frac{K(1-\frac{M}{N})}{1+\frac{KM}{N}}$. It was shown that $T_{max}$ is approximately optimal when $\alpha =\alpha^{c}$ and $\beta =\beta^{c}$, where

$$\alpha^{c}=\beta^{c}=\min\left(\frac{2K_{1}K_{2}M_{1}M_{2}+M_{1}N+K_{1}M_{1}N}{2K_{1}K_{2}M_{2}N-2K_{2}M_{2}N},1\right).$$

Zhang et al. [21] improved the scheme in [19] by jointly designing the data placement and delivery in two layers, effectively avoiding the transmission of any content already stored by the users. On this basis, Kong et al. [22] defined a combinatorial structure called a hierarchical placement delivery array (HPDA) to design hierarchical coded caching schemes, and proposed a transformation from two PDAs to an HPDA. When the two PDAs are chosen from the MN PDA, the resulting scheme is shown in Lemma 1, which aligns with the centralized scheme in [23], and achieves a smaller $R_{1}$ and the same $R_{2}$ compared to the scheme in [21]. Based on the definition of HPDA, Rashid Ummer et al. [24] designed two hierarchical coded caching schemes via t-designs. In addition, Pandey et al. [25] considered a hierarchical coded caching problem with coded placement. A wireless scenario was considered in [26,27], where each mirror connects to users via a wireless channel.

**Lemma** **1** ([22])**.** *When $\frac{M_{1}}{N}\in \left\{0,\frac{1}{K_{1}},\cdots ,\frac{K_{1}-1}{K_{1}},1\right\}$ and $\frac{M_{2}}{N}\in \left\{0,\frac{1}{K_{2}},\cdots ,\frac{K_{2}-1}{K_{2}},1\right\}$, there exists a $\left(K_{1},K_{2},M_{1},M_{2},N\right)$ hierarchical coded caching scheme with transmission loads $R_{1}=r_{c}\left(M_{1},K_{1}\right)\cdot r_{c}\left(M_{2},K_{2}\right),\;R_{2}=r_{c}\left(M_{2},K_{2}\right)$, where $r_{c}\left(M,K\right)=\frac{K(1-\frac{M}{N})}{1+\frac{KM}{N}}$.*

### 1.2. Contribution and Organization

In this paper, we consider the ($K_{1},K_{2},M_{1},M_{2},N$) two-layer hierarchical network proposed in [19]. Most existing schemes focus on creating as many multicast opportunities as possible among users connected to the same mirror, thus reducing the load from the mirror to its connected users (i.e., $R_{2}$), without fully leveraging the mirror and user caches to create multicast opportunities among all users. We aim to design a coded caching scheme for a two-layer hierarchical network to further reduce the coding delays $T_{max}$ and $T_{sum}$. To this end, we first treat the mirrors and users as cache nodes of the same type, i.e., the cache memory of each mirror is regarded as an additional part of its connected users’ cache, then the server broadcasts messages to all mirrors according to a $K_{1}K_{2}$-user MN PDA in the first layer. In the second layer, each mirror first cancels the useless packets (if any) in the received useful messages and forwards them to its connected users, such that each user can decode the requested packets not cached by the mirror, then broadcasts coded subpackets to its connected users according to a $K_{2}$-user MN PDA, such that each user can decode the requested packets cached by the mirror. The proposed scheme is extended to a heterogeneous two-layer hierarchical network, where the number of users connected to each mirror is different. Performance analysis showed that both the achieved coding delays $T_{max}$ and $T_{sum}$ of the proposed scheme are lower than those of existing schemes.

The rest of this paper is organized as follows: Section 2 introduces the definition of PDA and related results. Section 3 shows the main results of this paper. Section 4 presents an illustrative example. Performance analysis is provided in Section 5, and Section 6 concludes this paper.

*Notations:*

- For any integers *a* and *b* with a<b, we define [a,b]≜{a,a+1,⋯,b} and [a,b)≜{a,a+1,⋯,b−1}.
- For any positive integers *c*, we define [c]≜{1,2,⋯,c}.
- For two integers x,y, if 0≤y≤x, xy is the binomial coefficient defined as xy=x!y!(x−y)!, and we let xy=0 if x≤0 or x<y or y<0.
- For any F×K array P, P(i,j) represents the element in the *i*-th row and *j*-th column of P, where i∈[F] and j∈[K].

To improve readability, we add Table 1 to summarize frequently used symbols.

## 2. Placement Delivery Array

This section reviews the definition of PDA and the relationship between a PDA and a coded caching scheme.

**Definition** **1** ([7])**.** *For any positive integers K, F, Z, S, an F×K array P with alphabet {∗}∪[S] is called a (K,F,Z,S) placement delivery array (PDA) if it satisfies the following conditions:*

**C1.**  *The symbol “∗” appears Z times in each column;*
**C2.**  *Each integer s∈[S] occurs at least once in the array;*
**C3.**  *For any two distinct entries $P(j_{1},k_{1})$ and $P(j_{2},k_{2})$, if $P\left(j_{1},k_{1}\right)=P\left(j_{2},k_{2}\right)=s$, then $j_{1}\neq j_{2},k_{1}\neq k_{2}$ and $P\left(j_{1},k_{2}\right)=P\left(j_{2},\;k_{1}\right)=*$.*

*If each integer occurs exactly g times in a PDA, the PDA is called a g-regular PDA or g-PDA.*

In a PDA, the symbol “∗” in the *j*-th row and *k*-th column indicates that the *k*-th user stores the *j*-th packet of all files in the placement phase. The integer in the *j*-th row and *k*-th column indicates that the *k*-th user does not store the *j*-th packet of all files. The requested packets represented by the same integer in the PDA are sent to the users after the xoring operation in the delivery phase. Condition **C1** implies that the memory ratio of each user is Z/F. Condition **C3** ensures that each user can decode its requested packet, since it has cached all the other packets in the received coded message. Condition **C2** implies that the number of coded messages broadcast by the server is exactly *S*, each of size 1/F file, so the transmission load is R=S/F.

**Lemma** **2** ([7])**.** *Given a (K,F,Z,S) PDA, there exists a (K,M,N) coded caching scheme with user memory ratio $\frac{M}{N}=\frac{Z}{F}$, subpacketization F and transmission load $R=\frac{S}{F}$.*

When $t=\frac{KM}{N}\in \left[0,K\right)$, the MN PDA corresponding to the MN scheme is defined as follows: for any k∈[K] and for any T⊆[K] with |T|=t,

$$P\left(T,k\right)=\left\{\begin{array}{cc} {}* & \mathrm{if}\;k\in T,\\ f_{t+1}\left(T\cup \left\{k\right\}\right) & \mathrm{if}\;k\notin T, \end{array}\right.$$

where $f_{t+1}$ is an injection from {S|S⊆[K],|S|=t+1} to Kt+1. For example, when K=4 and $\frac{M}{N}=\frac{1}{2}$, the MN PDA is as follows:

$$P=\left[\begin{array}{cccc} {}* & * & 1 & 2\\ {}* & 1 & * & 3\\ {}* & 2 & 3 & *\\ 1 & * & * & 4\\ 2 & * & 4 & *\\ 3 & 4 & * & * \end{array}\right].$$

**Lemma** **3** ([7])**.** *For any positive integers K and t with t<K, the MN PDA is a (t+1)-K,Kt,K−1t−1,Kt+1 PDA.*

## 3. Main Results

In this section, we propose a coded caching scheme for a two-layer hierarchical network using two MN PDAs. The first MN PDA is for all the $K_{1}K_{2}$ users, which fully utilizes the cache memory of all mirrors and users to create multicast opportunities among all users. The second MN PDA is for each group of $K_{2}$ users connected to the same mirror, which fully utilizes user caches to create multicast opportunities within each group. The main result is as follows:

**Theorem** **1.** *In the $\left(K_{1},K_{2},M_{1},M_{2},N\right)$ two-layer hierarchical network, let $K=K_{1}K_{2}$, for any $\mu \in [K_{2},K]$, $t\in [0,K_{2}]$, there exists a coded caching scheme with*

(1) M1N=K−K2μ−K2Kμ,M2N=μK−(1−tK2)K−K2μ−K2Kμ,

(2) R1=K−μμ+1,R2=Kμ+1−K−K2μ+1Kμ+K−K2μ−K2KμK2−tt+1.

**Proof.**  For any $\mu \in [K_{2},K]$ and $t\in [0,K_{2}]$, there is a (μ+1)-(K,F,Z,S) MN PDA G and a (t+1)-$\left(K_{2},F^{\prime },Z^{\prime },S^{\prime }\right)$ MN PDA P, where F=Kμ, Z=K−1μ−1, S=Kμ+1, F′=K2t, Z′=K2−1t−1, S′=K2t+1. The PDA G is divided into $K_{1}$ subarrays, i.e., $G=[G_{1},\cdots ,G_{K_{1}}]$, where $G_{k_{1}}$ contains $K_{2}$ columns for each $k_{1}\in \left[K_{1}\right]$. The hierarchical coded caching scheme in Theorem 1 is generated based on the PDA G and P as follows:

- **Placement Phase:** Each file $W_{i}$ is split into *F* packets, i.e., $W_{i}=\left\{W_{i,j}|j\in \left[F\right]\right\}$. The *j*-th packet of each file is cached by the $k_{1}$-th mirror if each element of the *j*-th row in the subarray $G_{k_{1}}$ is “∗”, i.e.,
  (3) $$M_{k_{1}}=\left\{W_{i,j}|\forall k_{2}\in \left[K_{2}\right],G_{k_{1}}\left(j,k_{2}\right)=*,i\in \left[N\right]\right\}.$$
  Since the number of rows in each $G_{k_{1}}$ consisting entirely of “∗”s is K−K2μ−K2, the memory ratio of each mirror is
  M1N=K−K2μ−K2F=K−K2μ−K2Kμ.
  The cached content of user $\left(k_{1},k_{2}\right)$ consists of two parts, i.e.,
  (4) $$U_{\left(k_{1},k_{2}\right)}=U_{\left(k_{1},k_{2}\right)}^{1}\cup U_{\left(k_{1},k_{2}\right)}^{2},$$
  where $U_{\left(k_{1},k_{2}\right)}^{1}$ is not cached by the $k_{1}$-th mirror and $U_{\left(k_{1},k_{2}\right)}^{2}$ is a subset of the content cached by the $k_{1}$-th mirror. Specifically, the *j*-th packet of each file is cached by user $\left(k_{1},k_{2}\right)$ if it is not cached by the $k_{1}$-th mirror and the element at the *j*-th row and $k_{2}$-th column of the subarray $G_{k_{1}}$ is “∗”, i.e.,
  (5) $$U_{\left(k_{1},k_{2}\right)}^{1}=\left\{W_{i,j}|G_{k_{1}}\left(j,k_{2}\right)=*,\;W_{i,j}\notin M_{k_{1}},\;i\in \left[N\right]\right\}.$$
  Each packet cached by the $k_{1}$-th mirror is further divided into $F^{\prime }$ subpackets, i.e., for any $W_{i,j}\in M_{k_{1}}$, we have $W_{i,j}=\left\{W_{i,j,h}|h\in \left[F^{\prime }\right]\right\}$. The subpacket $W_{i,j,h}$ is cached by user $\left(k_{1},k_{2}\right)$ if the corresponding packet $W_{i,j}$ is cached by the $k_{1}$-th mirror and the element at the *h*-th row and $k_{2}$-th column of P is “∗”, i.e.,
  (6) $$U_{\left(k_{1},k_{2}\right)}^{2}=\left\{W_{i,j,h}|W_{i,j}\in M_{k_{1}},\;P\left(h,k_{2}\right)=*,\;i\in \left[N\right]\right\}.$$
  Hence, the memory ratio of each user is
  M2N=Z−K−K2μ−K2F+M1N·Z′F′=μK−(1−tK2)K−K2μ−K2Kμ.
- **Delivery Phase:** Each user requests a file from the server, assuming that user $\left(k_{1},k_{2}\right)$ requests the file $W_{d_{\left(k_{1},k_{2}\right)}}$. The transmission from the server to the mirrors is according to the PDA G. Specifically, for any integer s∈[S], the server sends
  (7) Xs=⨁G(j,k)=s,j∈[F],k∈[K],k1=⌈kK2⌉,k2=k−(k1−1)·K2Wd(k1,k2),j
  to the mirrors. Therefore, the transmission load from the server to the mirrors is
  $$R_{1}=\frac{S}{F}=\frac{K-\mu }{\mu +1}.$$
  The transmission from each mirror to the attached users consists of two parts. First, each mirror cancels useless packets (if any) in the received useful messages using the cached content, then forwards them to the attached users. The number of messages forwarded by each mirror is Kμ+1−K−K2μ+1. Second, each mirror transmits its cached contents to its attached users according to the PDA P. Specifically, for any packet index *j* satisfying that the *j*-th packet of each file is cached by the $k_{1}$-th mirror, i.e., $W_{i,j}\in M_{k_{1}}$ where i∈[N], for any integer $s\in [S^{\prime }]$, the $k_{1}$-th mirror sends
  (8) $$Y_{s,j}^{k_{1}}=\underset{P\left(h,k_{2}\right)=s,h\in \left[F^{\prime }\right],k_{2}\in \left[K_{2}\right],}{⨁}W_{d_{\left(k_{1},k_{2}\right)},j,h}$$
  to all its attached users. Hence, the transmission load from each mirror to its attached users is
  R2=Kμ+1−K−K2μ+1F+K−K2μ−K2S′F·F′=Kμ+1−K−K2μ+1Kμ+K−K2μ−K2KμK2−tt+1.

By using the messages forwarded by the $k_{1}$-th mirror, user $\left(k_{1},k_{2}\right)$ can recover each requested packet not cached by the $k_{1}$-th mirror, since G is a PDA. By using the messages in (8), user $\left(k_{1},k_{2}\right)$ can recover each requested packet cached by the $k_{1}$-th mirror, since P is also a PDA. Hence, each user can recover its desired file. □

The scheme in Theorem 1 can be extended to a heterogeneous scenario where the number of users connected to each mirror may vary. Precisely, there is a central server containing *N* files connected to *A* mirrors. For any i∈[A], there are $L_{i}$ users connected to the *i*-th mirror. For any $j\in [L_{i}]$, the *j*-th user connected to the *i*-th mirror (denoted by user (i,j)) has a cache memory of size $M_{\left(i,j\right)}$ files and the *i*-th mirror has a cache memory of size $M_{i}$ files. This scenario is called a heterogeneous two-layer hierarchical network.

For the heterogeneous two-layer hierarchical network, we choose a (K,F,Z,S) MN PDA G, where $K=\sum_{i=1}^{A}L_{i}$, F=Kμ, Z=K−1μ−1, S=Kμ+1, and $\mu \in [\max_{i\in [A]}\left(L_{i}\right),K]$. G is divided into *A* subarrays, i.e., $G=[G_{1},\cdots ,G_{A}]$, where $G_{i}$ contains $L_{i}$ columns. For any i∈[A], we choose an $\left(L_{i},F_{i},Z_{i},S_{i}\right)$ MN PDA $P_{i}$ where Fi=Liti, Zi=Li−1ti−1, Si=Liti+1 and $t_{i}\in \left[0,L_{i}\right]$.

In the placement phase, each file $W_{n}$ is split into *F* packets, i.e., $W_{n}=\left\{W_{n,f}|f\in \left[F\right]\right\}$. The *f*-th packet of each file is cached by the *i*-th mirror if the *f*-th row of the subarray $G_{i}$ consists entirely of “∗”s. Then, the memory ratio of the *i*-th mirror is

MiN=K−Liμ−LiKμ,i∈[A].

The cached content of user (i,j) includes two parts. The first part is completely non-cached by the i-th mirror. That is, the *f*-th packet of each file is cached by user (i,j) if it is not cached by the *i*-th mirror and $G_{i}\left(f,j\right)=*$. The second part is a subset of the content cached by the *i*-th mirror. That is, each packet $W_{n,f}$ cached by the *i*-th mirror is further divided into $F_{i}$ subpackets, i.e., $W_{n,f}=\left\{W_{n,f,h}|h\in \left[F_{i}\right]\right\}$, and the *h*-th subpacket of $W_{n,f}$ is stored by user (i,j) if $P_{i}\left(h,j\right)=*$. Therefore, the total memory ratio of user (i,j) is

M(i,j)N=K−1μ−1−K−Liμ−LiKμ+K−Liμ−LiKμ·tiLi=μK−(1−tiLi)K−Liμ−LiKμ.

In the delivery phase, the server broadcasts *S* coded messages to the mirrors according to the PDA G, thus the transmission load from the server to the mirrors is

$$R_{1}=\frac{K-\mu }{\mu +1}.$$

For any i∈[A], the *i*-th mirror first cancels useless packets (if any) in the received useful messages by using cached content and forwards them to the attached users. The number of messages forwarded by the *i*-th mirror is Kμ+1−K−Liμ+1. Then, the *i*-th mirror broadcasts $S_{i}$ coded subpackets to the attached users according to $P_{i}$. Hence, the total transmission load from the *i*-th mirror to its connected users is

R2i=Kμ+1−K−Liμ+1Kμ+K−Liμ−LiKμLi−titi+1.

Since G and $P_{i}\left(i\in \left[A\right]\right)$ are all PDAs, each user can decode its desired file. The following result is obtained:

**Theorem** **2.** *For the heterogeneous two-layer hierarchical network, there exists a coded caching scheme with the memory ratio of the i-th mirror*

MiN=K−Liμ−LiKμ,

*the memory ratio of the j-th user connected to the i-th mirror*

M(i,j)N=μK−(1−tiLi)K−Liμ−LiKμ,

*the transmission load from the server to the mirrors*

$$R_{1}=\frac{K-\mu }{\mu +1},$$

*and the transmission load from the i-th mirror to the attached users*

R2i=Kμ+1−K−Liμ+1Kμ+K−Liμ−LiKμLi−titi+1,

*where i∈[A], $j\in [L_{i}]$, $K=\sum_{i=1}^{A}L_{i}$, $\mu \in [\max_{i\in [A]}\left(L_{i}\right),K]$ and $t_{i}\in \left[0,L_{i}\right]$.*

Note that when $A=K_{1}$, $L_{1}=L_{2}=\cdots =L_{A}=K_{2}$ and $t_{1}=t_{2}=\cdots =t_{A}=t$, the scheme in Theorem 2 reduces to the scheme in Theorem 1.

## 4. An Illustrative Example for Theorem 1

For the ($K_{1},K_{2},M_{1},M_{2},N$) two-layer hierarchical network where $K_{1}=2,\;K_{2}=2,\;M_{1}/N=1/6,\;M_{2}/N=5/12$, we have $K=K_{1}K_{2}=4$. By choosing μ=2, the (K,F,Z,S) MN PDA G is shown in (9), where F=6, Z=3, and S=4. G is divided equally into $K_{1}=2$ subarrays, each with $K_{2}=2$ columns, i.e., $G=[G_{1},G_{2}]$.

(9) $$G=\begin{array}{c} G_{1}\;G_{2}\\ \left[\begin{array}{cccc} {}* & * & 1 & 2\\ {}* & 1 & * & 3\\ {}* & 2 & 3 & *\\ 1 & * & * & 4\\ 2 & * & 4 & *\\ 3 & 4 & * & * \end{array}\right]. \end{array}$$

By choosing t=1, the $\left(K_{2},F^{\prime },Z^{\prime },S^{\prime }\right)$ MN PDA P is shown in (10), where $F^{\prime }=2$, $Z^{\prime }=1$, and $S^{\prime }=1$.

(10) $$P=\left[\begin{array}{cc} {}* & 1\\ 1 & * \end{array}\right].$$

The placement and delivery phases of the hierarchical coded caching scheme are generated by the PDA G in (9) and P in (10), as follows:

- **Placement Phase:** Each file $W_{i}$ is split into F=6 packets, i.e., $W_{i}=\left\{W_{i,j}|j\in \left[6\right]\right\}$. The *j*-th packet of each file is cached by the $k_{1}$-th mirror if each element of the *j*-th row in the subarray $G_{k_{1}}$ is “∗”. From (3) and (9), the cached content of each mirror is as follows:
  (11) $$M_{1}=\left\{W_{i,1}|i\in \left[N\right]\right\},\;\;\;M_{2}=\left\{W_{i,6}|i\in \left[N\right]\right\}.$$
  thus, the memory ratio of each mirror is $\frac{M_{1}}{N}=\frac{1}{6}$.
  The cached content of user $\left(k_{1},k_{2}\right)$ consists of two parts, i.e., $U_{\left(k_{1},k_{2}\right)}=U_{\left(k_{1},k_{2}\right)}^{1}\cup U_{\left(k_{1},k_{2}\right)}^{2}$, where the first part $U_{\left(k_{1},k_{2}\right)}^{1}$ is not cached by the $k_{1}$-th mirror, and the second part $U_{\left(k_{1},k_{2}\right)}^{2}$ is a subset of the content cached by the $k_{1}$-th mirror. From (5) and (10), the first part of the cached content of each user is as follows:
  $$\begin{array}{c} U_{\left(1,1\right)}^{1}=\left\{W_{i,2},W_{i,3}|i\in \left[N\right]\right\},\;U_{\left(1,2\right)}^{1}=\left\{W_{i,4},W_{i,5}|i\in \left[N\right]\right\},\\ U_{\left(2,1\right)}^{1}=\left\{W_{i,2},W_{i,4}|i\in \left[N\right]\right\},\;U_{\left(2,2\right)}^{1}=\left\{W_{i,3},W_{i,5}|i\in \left[N\right]\right\}. \end{array}$$
  Each packet cached by the $k_{1}$-th mirror is further divided into $F^{\prime }=2$ subpackets, i.e., for any $W_{i,j}\in M_{k_{1}}$, we have $W_{i,j}=\left\{W_{i,j,h}|h\in \left[2\right]\right\}$. From (6), (10) and (11), the second part of the cached content of each user is as follows:
  $$\begin{array}{c} U_{\left(1,1\right)}^{2}=\left\{W_{i,1,1}|i\in \left[N\right]\right\},\;\;\;U_{\left(1,2\right)}^{2}=\left\{W_{i,1,2}|i\in \left[N\right]\right\},\\ U_{\left(2,1\right)}^{2}=\left\{W_{i,6,1}|i\in \left[N\right]\right\},\;\;\;U_{\left(2,2\right)}^{2}=\left\{W_{i,6,2}|i\in \left[N\right]\right\}. \end{array}$$
  Hence, the memory ratio of each user is $\frac{M_{2}}{N}=\frac{2}{6}+\frac{1}{6}\cdot \frac{1}{2}=\frac{5}{12}$.
- **Delivery Phase:** Each user requests a file from the server, assuming the request vector is
  $$d=\left(d_{\left(1,1\right)},d_{\left(1,2\right)},d_{\left(2,1\right)},d_{\left(2,2\right)}\right)=\left(1,2,3,4\right),$$
  i.e., user (1,1),(1,2),(2,1),(2,2) request $W_{1},W_{2},W_{3},W_{4}$ respectively. The transmission from the server to the mirrors is according to the PDA G in (9). Specifically, the server sends
  $$\begin{array}{c} X_{1}=W_{1,4}⊕W_{2,2}⊕W_{3,1},\;\;\;X_{2}=W_{1,5}⊕W_{2,3}⊕W_{4,1},\\ X_{3}=W_{1,6}⊕W_{3,3}⊕W_{4,2},\;\;\;X_{4}=W_{2,6}⊕W_{3,5}⊕W_{4,4} \end{array}$$
  to the mirrors from (7) and (9). Therefore, the transmission load from the server to the mirrors is $R_{1}=\frac{4}{6}=\frac{2}{3}$.
  The transmission from each mirror to the attached users consists of two parts. First, each mirror cancels useless packets (if any) in the received useful messages by using the cached content, then forwards them to the attached users. Specifically, the first mirror cancels $W_{3,1}$ in $X_{1}$ to obtain $X_{1}^{1}=W_{1,4}⊕W_{2,2}$, cancels $W_{4,1}$ in $X_{2}$ to obtain $X_{2}^{1}=W_{1,5}⊕W_{2,3}$, then forwards $X_{1}^{1},X_{2}^{1},X_{3},X_{4}$ to user (1,1) and (1,2). The second mirror cancels $W_{1,6}$ in $X_{3}$ to obtain $X_{3}^{2}=W_{3,3}⊕W_{4,2}$, cancels $W_{2,6}$ in $X_{4}$ to obtain $X_{4}^{2}=W_{3,5}⊕W_{4,4}$, then forwards $X_{1},X_{2},X_{3}^{2},X_{4}^{2}$ to user (2,1) and (2,2). Second, each mirror transmits coded subpackets to the attached users according to the PDA P in (10). Specifically, the first mirror sends
  $$Y_{1,1}^{1}=W_{1,1,2}⊕W_{2,1,1}$$
  to its attached users, and the second mirror sends
  $$Y_{1,6}^{2}=W_{3,6,2}⊕W_{4,6,1}$$
  to its attached users from (8), (10) and (11). Thus, the transmission load from each mirror to its attached users is $R_{2}=\frac{4}{6}+\frac{1}{12}=\frac{5}{12}$.

After receiving the messages from the connected mirror, each user can recover their desired file. For example, let us consider user (1,1) who requests the file $W_{1}=\left\{W_{1,j}|j\in \left[6\right]\right\}$. First, it can decode $W_{1,4}$ from $X_{1}^{1}$, since it caches $W_{2,2}$. It can decode $W_{1,5}$ from $X_{2}^{1}$, since it caches $W_{2,3}$. It can decode $W_{1,6}$ from $X_{3}$, since it caches $W_{3,3}$ and $W_{4,2}$. Second, it can obtain subpacket $W_{1,1,2}$ from $Y_{1,1}^{1}$, since it caches $W_{i,1,1}$ where i∈[N], thus obtaining the packet $W_{1,1}$. The remaining packets $W_{1,2}$ and $W_{1,3}$ are cached by user (1,1). Thus, user (1,1) can recover the desired file $W_{1}$. The decodability for other users is similar.

## 5. Performance Analysis

**Figure 2:**
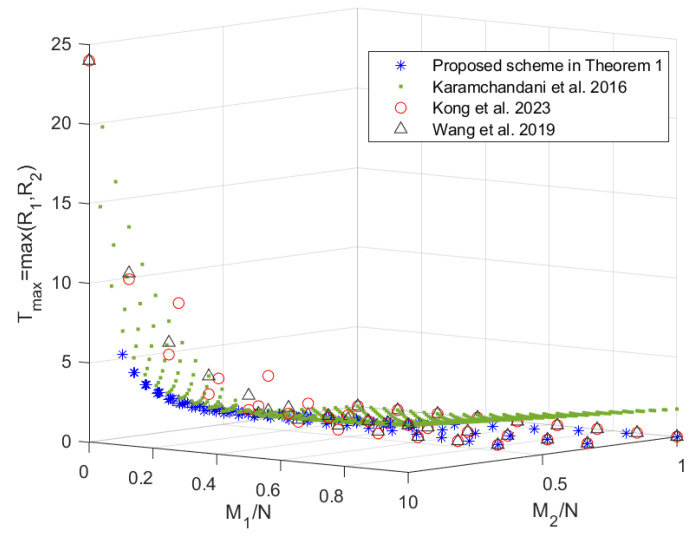
The tradeoff of the coding delay $T_{max}$ with the memory ratios of each mirror and user of the proposed scheme and the schemes in [19,20,22] when $K_{1}=8,K_{2}=3$.

**Figure 3:**
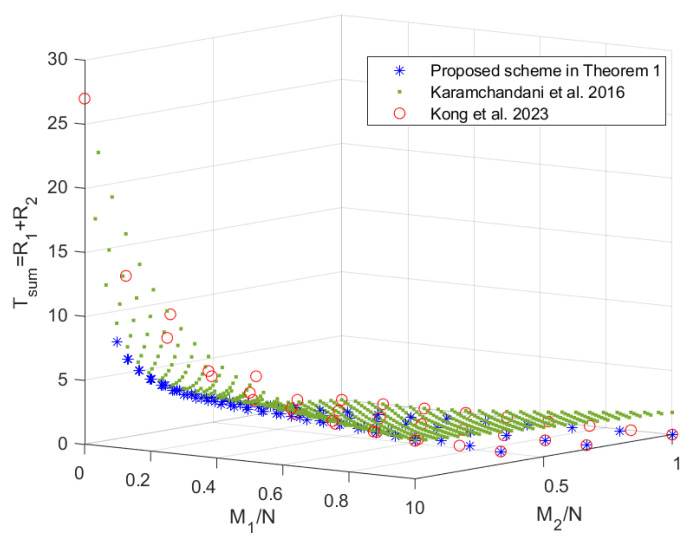
The tradeoff of the coding delay $T_{sum}$ with the memory ratios of each mirror and user of the proposed scheme and the schemes in [19,22] when $K_{1}=8,K_{2}=3$.

**Figure 4:**
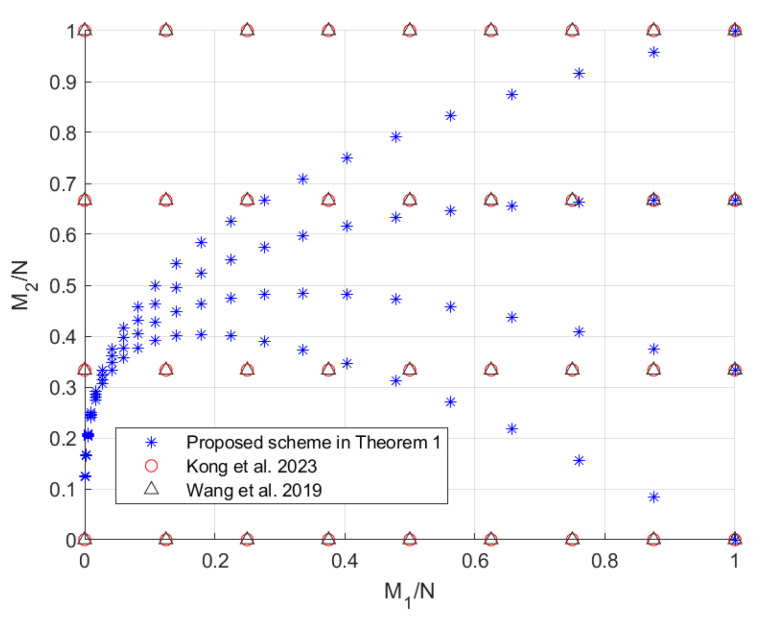
The applicable memory ratio points for the schemes in [20,22] and the scheme in Theorem 1 when $K_{1}=8,K_{2}=3$.

**Figure 5:**
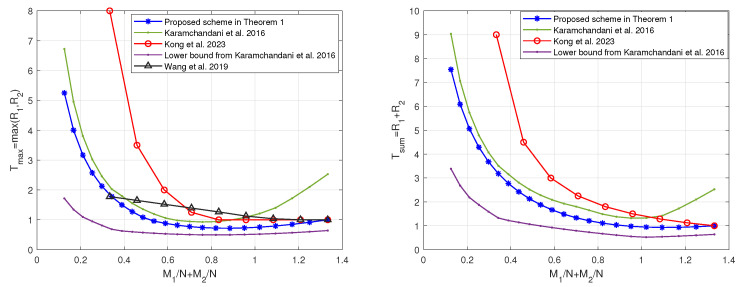
The tradeoffs of the coding delays with the sum of memory ratios of the proposed scheme and the schemes in [19,20,22] when $K_{1}=8$ and $K_{2}=3$.

In this section, we compare the scheme in Theorem 1 with the schemes in [19,20,22], since the scheme in [24] does not provide an exact expression for $R_{2}$ and the scheme in [22] (which is consistent with the centralized scheme in [23]) outperforms the scheme in [21]. When $K_{1}=8>K_{2}=3$, the tradeoffs of the coding delays $T_{max},T_{sum}$ with the memory ratios of each mirror and user, i.e., $\frac{M_{1}}{N}$ and $\frac{M_{2}}{N}$, are shown in Figure 2 and Figure 3, respectively. Note that the scheme in [20] only focuses on the maximum coding delay $T_{max}$. The scheme in [19] is applicable to arbitrary memory ratios, while the scheme proposed in Theorem 1 and the schemes in [20,22] are applicable to specific memory ratios, which are shown in Figure 4. It can be seen from Figure 2 and Figure 3 that the scheme in Theorem 1 achieves lower coding delays $T_{max}$ and $T_{sum}$ than the schemes in [19,20,22]. To see this more clearly, we restrict the scheme in [19] to the memory ratio points applicable to the scheme in Theorem 1, and the obtained tradeoffs of the coding delays $T_{max}$ and $T_{sum}$ with the sum of memory ratios $\frac{M_{1}}{N}+\frac{M_{2}}{N}$ are shown in Figure 5. It can be seen that under the same sum of memory ratios, both the coding delays $T_{max}$ and $T_{sum}$ of the scheme in Theorem 1 are lower than those of the schemes in [19,20,22]. Moreover, when the sum of memory ratios is relatively large or small, the two coding delays of the scheme in Theorem 1 are significantly reduced compared to the scheme in [19]. This is primarily due to the fact that, in the first layer of communication, we have fully leveraged the caches of both the mirrors and the users to create as many multicast opportunities as possible among all users; in the second layer of communication, we once again make full use of the users’ caches to maximize multicast opportunities within each group of users connected to the same mirror.

**Figure 6:**
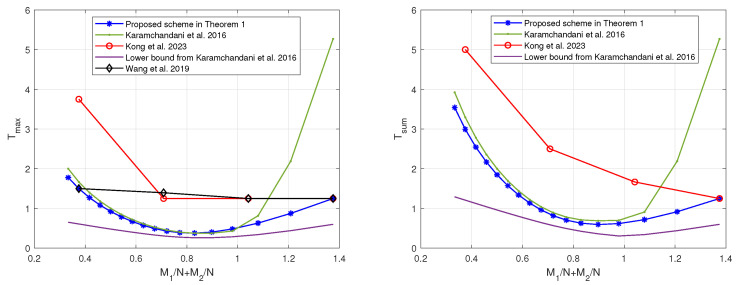
The tradeoffs of the coding delays with the sum of memory ratios of the proposed scheme and the schemes in [19,20,22] when $K_{1}=3$ and $K_{2}=8$.

**Figure 7:**
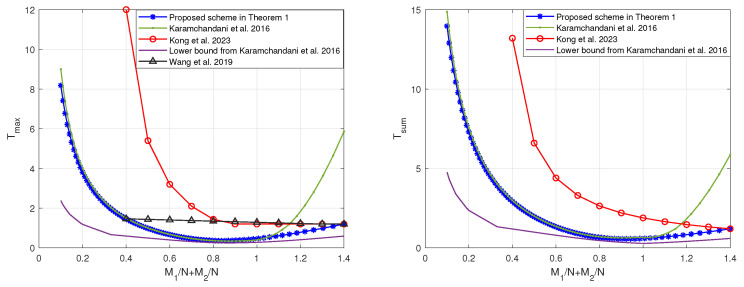
The tradeoffs of the coding delays with the sum of memory ratios of the proposed scheme and the schemes in [19,20,22] when $K_{1}=10$ and $K_{2}=10$.

Similarly, when $K_{1}=3<K_{2}=8$ or $K_{1}=K_{2}=10$, by restricting the scheme in [19] to the memory ratio points that are compatible with the scheme in Theorem 1, the tradeoffs of the coding delays $T_{max}$ and $T_{sum}$ with the sum of memory ratios $\frac{M_{1}}{N}+\frac{M_{2}}{N}$ are as shown in Figure 6 and Figure 7, respectively. In can be seen that, regardless of the relative sizes of $K_{1}$ and $K_{2}$, our proposed scheme is capable of achieving reduced coding delays across a majority of memory ratio points.

## 6. Conclusions

In this paper, we proposed a novel coded caching scheme for the two-layer hierarchical network by utilizing two MN PDAs. The first MN PDA is for all users, which fully utilizes the cache memory of all mirrors and users to create as many multicast opportunities as possible. The second MN PDA is for a group of users connected to the same mirror, which fully utilizes the user’s cache to create multicast opportunities within the group. Moreover, the proposed scheme was extended to a heterogeneous two-layer hierarchical network. Numerical comparisons showed that the proposed scheme achieved lower coding delays $T_{max}$ and $T_{sum}$ than existing schemes in [19,20,22] at most memory ratio points.

## Figures and Tables

**Table 1:** Table of parameters.

Notations or Acronyms	Meaning
$K_{1}$	The number of mirrors
$K_{2}$	The number of users connected to each mirror
$K=K_{1}K_{2}$	The total number of users
*N*	The number of files
$M_{1}$	The cache size of each mirror
$M_{2}$	The cache size of each user
$R_{1}$	The communication load from the server to the mirrors
$R_{2}$	The communication load from each mirror to the attached users
$T_{max}=max\left(R_{1},R_{2}\right)$	The maximum coding delay
$T_{sum}=R_{1}+R_{2}$	The sum coding delay
$W_{i}$	The *i*-th file
$W_{i,j}$	The *j*-th packet of the *i*-th file
$W_{i,j,h}$	The *h*-th subpacket of the packet $W_{i,j}$
$M_{k_{1}}$	The cached content of the $k_{1}$-th mirror
$\left(k_{1},k_{2}\right)$	The $k_{2}$-th user connected to the $k_{1}$-th mirror
$W_{d_{\left(k_{1},k_{2}\right)}}$	The file requested by user $\left(k_{1},k_{2}\right)$
$U_{\left(k_{1},k_{2}\right)}$	The cached content of user $\left(k_{1},k_{2}\right)$
$U_{\left(k_{1},k_{2}\right)}^{1}$	The cached content of user $\left(k_{1},k_{2}\right)$ which is not cached by the $k_{1}$-th mirror
$U_{\left(k_{1},k_{2}\right)}^{2}$	The cached content of user $\left(k_{1},k_{2}\right)$ which is cached by the $k_{1}$-th mirror
PDA	Placement delivery array
MN PDA	The PDA corresponding to the MN scheme in [1]

## Data Availability

The data are available upon request from the corresponding author.
